# Metal Ions, Not Metal-Catalyzed Oxidative Stress, Cause Clay Leachate Antibacterial Activity

**DOI:** 10.1371/journal.pone.0115172

**Published:** 2014-12-11

**Authors:** Caitlin C. Otto, Jennifer L. Koehl, Dipesh Solanky, Shelley E. Haydel

**Affiliations:** 1 School of Life Sciences, Arizona State University, Tempe, Arizona, United States of America; 2 The Biodesign Institute Center for Infectious Diseases and Vaccinology, Arizona State University, Tempe, Arizona, United States of America; Louisiana State University Health Sciences Center, United States of America

## Abstract

Aqueous leachates prepared from natural antibacterial clays, arbitrarily designated CB-L, release metal ions into suspension, have a low pH (3.4–5), generate reactive oxygen species (ROS) and H_2_O_2_, and have a high oxidation-reduction potential. To isolate the role of pH in the antibacterial activity of CB clay mixtures, we exposed three different strains of *Escherichia coli* O157:H7 to 10% clay suspensions. The clay suspension completely killed acid-sensitive and acid-tolerant *E. coli* O157:H7 strains, whereas incubation in a low-pH buffer resulted in a minimal decrease in viability, demonstrating that low pH alone does not mediate antibacterial activity. The prevailing hypothesis is that metal ions participate in redox cycling and produce ROS, leading to oxidative damage to macromolecules and resulting in cellular death. However, *E. coli* cells showed no increase in DNA or protein oxidative lesions and a slight increase in lipid peroxidation following exposure to the antibacterial leachate. Further, supplementation with numerous ROS scavengers eliminated lipid peroxidation, but did not rescue the cells from CB-L-mediated killing. In contrast, supplementing CB-L with EDTA, a broad-spectrum metal chelator, reduced killing. Finally, CB-L was equally lethal to cells in an anoxic environment as compared to the aerobic environment. Thus, ROS were not required for lethal activity and did not contribute to toxicity of CB-L. We conclude that clay-mediated killing was not due to oxidative damage, but rather, was due to toxicity associated directly with released metal ions.

## Introduction

We have identified a natural clay mixture, arbitrarily termed CB, that displays broad-spectrum, in vitro bactericidal activity in hydrated suspensions [1]–[4]. Previously, we prepared aqueous extracts from the natural clay mixtures, termed leachates, that are devoid of physical particles. These leachates, designated CB-L, maintain in vitro antibacterial activity, but do not cause cell lysis [2]. Thus, killing is dependent on the abiotic chemical, not physical, properties of hydrated clay suspensions [1]–[3]. When in hydrated suspension, the pH range of CB clay mixtures is 3–4 [1]. While we have previously shown that CB killing activity is dependent on a low pH environment, the acidic environment generated by CB minerals does not solely mediate toxicity [3]. Moreover, supplementation of a non-antibacterial leachate containing lower concentrations of Fe, Co, Ni, Cu, and Zn to final ion concentrations and a pH equivalent to that of the antibacterial leachate generated antibacterial activity against *Escherichia coli* and methicillin-resistant *Staphylococcus aureus* (MRSA), confirming the role of these ions in the antibacterial clay mixture leachates [1].

The first goal of the present study was to determine whether the acidic environment itself is exerting a degree of toxicity or whether it is simply necessary to maintain the desorbed metal ions in their more toxic speciation. For example, Fe is more toxic in acidic environments due to the increased solubility and reactivity of its reduced form, whereas Cu, Zn, and Cd are more toxic in elevated pH environments due to less competition with protons for cellular binding sites and less efflux [5]–[7]. *E. coli* O157:H7 was used as the model organism during low pH experimentation as specific strains offer extreme acid resistance resulting from cross protection between three well-characterized acid-response systems [8], [9]. The first acid-resistance system (AR1) is induced upon entry into stationary phase and involves activation of the RpoS general stress response [10]–[12]. AR2 transports glutamate into the cell via the antiporter GadC, converts glutamate to γ-amino butyric acid (GABA) by glutamate decarboxylase (GadA and GadB), and transports GABA out of the cell via GadC. During this continuous glutamate-to-GABA conversion, intracellular protons are consumed, thus raising the cytoplasmic pH and creating a pH gradient [13]–[15]. The second (AR2) and third (AR3) acid-resistance systems are functionally similar to one another, allowing the cytoplasmic pH to remain higher than the extracellular environment [11], [13], [14], [16], [17].

Chemical simulation modeling of the ions in CB-L revealed increased concentrations of soluble Cu^2+^ and Fe^2+^ in the antibacterial leachates, compared to non-antibacterial leachates, suggesting that these ionic species are modulating the antibacterial activity of the leachates [1]. Iron and copper are known to participate in the Fenton and Fenton-like reactions, respectively, whereby the metal reacts with H_2_O_2_ to produce a hydroxyl radical [18]. Current dogma asserts that reactive oxygen species (ROS)-driven oxidative damage to macromolecules is directly responsible for killing bacteria whereby H_2_O_2_ diffuses into the cells, damages DNA, and kills the cells [19], [20]. For example, Lloyd et al. [20] showed that salmon sperm DNA exposed to H_2_O_2_ along with at least one of nine different transition-metal ions showed significant increases in 8-hydroxydeoxyguanosine (8-OHdG), a biomarker of DNA damage. DNA damage has been shown to be iron-dependent, suggesting that the damage is actually from hydroxyl radicals or ferryl radical intermediates produced by the Fenton reaction [21], [22]. However, hydroxyl radicals are so reactive that they can only diffuse 5-10 molecular diameters before reacting nonspecifically with any reactive molecule in its vicinity [23]. Much of the current literature associated with ROS-driven oxidative damage to macromolecules is related directly to intracellular oxidative stress [24], [25]. Considering the high reactivity of hydroxyl radicals, superoxide, peroxynitrite, and hydrogen peroxide, oxidative damage to biological molecules is diffusion limited, and thus, exogenous ROS may not act on intracellular targets.

Here, we report evidence that metal-catalyzed oxidative damage is not responsible for antibacterial clay leachate-mediated killing. We also demonstrate that EDTA, not ROS scavengers or carbon supplements, disrupt antibacterial leachate killing and that bactericidal activity occurs in an anaerobic environment, thus negating the hypothesis that metal-catalyzed oxidative stress causes clay leachate antibacterial activity in vitro.

## Materials and Methods

### Bacterial Strains and Inoculum Preparation

*E. coli* ATCC 25922, *E. coli* O157:H7 ATCC 43889 (acid-tolerant), ATCC 43890 (acid-sensitive), and ATCC 43895 (acid-resistant, CDC EDL 933) were obtained from the American Type Culture Collection [26]. The methicillin-resistant *Staphylococcus aureus* (MRSA) strain, a clinical isolate obtained from Sonora Quest Laboratories (Tempe, AZ, USA), was grown as previously described [1]–[4]. For non-induced experiments, *E. coli* ATCC 25922 and *E. coli* O157:H7 were grown overnight in Luria-Bertani (LB) broth and trypticase soy broth (TSB), respectively, for 13–16 h at 37°C with gentle rotary mixing. Stationary-phase cultures were prepared by diluting saturated, overnight cultures into fresh growth medium to a starting cell density of 10^7^ or 10^8^ CFU/ml. Exponential phase cultures were prepared by diluting overnight cultures to a concentration of 10^7^ CFU/ml into fresh growth medium and continuing growth at 37°C until the cultures reached mid-logarithmic phase (OD_600_ 0.4–0.6). Prior to exposure, 1 ml aliquots of the cells were pelleted via centrifugation for 5 min at 13,000×*g*, washed with either 0.85% saline or 0.1× phosphate-buffered saline (PBS), pelleted via centrifugation for 5 min at 13,000×*g*, and suspended in the appropriate experimental medium. Non-induced stationary-phase *E. coli* O157:H7 (ATCC 43889) cells were suspended in EG medium, a minimal salts medium (73 mM K_2_HPO_4_, 17 mM NaNH_4_HPO_4_, 0.8 mM MgSO_4_) with 10 mM citrate and 0.4% glucose, and chemically-induced cells were suspended in EG medium with the addition of 2 mM glutamate [16]. All experiments were accompanied by cell viability assays, and CFU/ml was determined following overnight incubation at 37°C.

### Clay Mixtures and Clay Mixture Leachates

Clay mixture leachates were prepared as previously described [5], [6] with minor modifications. Briefly, CB-L was obtained by continuously stirring clay mixtures (1 g/10 ml) in sterile, ultrapure, deionized H_2_O (dH_2_O) for 18–24 h, followed by centrifugation (31,000×*g*) for 3 h at 4°C. The aqueous supernatant (leachate) was then sterilized by passage through a 0.22 µm filter.

### Anaerobic Incubations and Viability Assays

CB leachate and exponential phase *E. coli* ATCC 25922 cells were prepared as described above. Anaerobic dH_2_O and CB-L were prepared by sparging the solutions for 30 min with a steady stream (8 PSI) of compressed nitrogen (99.97% pure, Airgas, Riverside, CA) through a 1 L bottle of water at room temperature [27]. Bacterial cells were transferred to the anaerobic chamber, resuspended in the anaerobically-prepared solutions, and incubated under anoxic conditions at 37°C. Cell viability was determined by plating in duplicate on LB plates either directly from the experimental samples or following appropriate 10-fold dilutions at the specified times.

### pH Experiments

Initial screening experiments were performed to observe viability of acid-sensitive, non-induced acid-tolerant, and naturally acid-resistant *E. coli* O157:H7 when exposed to 10% suspensions of CB mixtures compared to a low-pH phosphate buffer. The pH of a 10% CB suspension is 3.1–3.3, so a low-pH 100 mM phosphate buffer (pH 3.1) was used for control experiments. Acid resistance was chemically induced in acid-tolerant *E. coli* O157:H7 by the addition of 2 mM glutamate to EG medium just prior to addition of 1% CB minerals. During acid-resistance induction, the pH of experimental media was adjusted to pH 2.5 with 1 M HCl.

### H_2_O_2_ Detection Assays

H_2_O_2_ measurements were performed using the Fluorescent Hydrogen Peroxide/Peroxidase Detection Kit following the manufacturer's protocol (Cell Technology, Inc., Mountain View, CA). Diluted cultures of stationary-phase *E. coli* ATCC 25922 (10^7^ CFU/ml) were prepared as described above and exposed to either a 10% CB mixture or CB-L. Briefly, after 0, 3, 6, and 9 h incubations, 150 µl aliquots were removed and centrifuged for 5 min at 13,000×*g*, and 50 µl of the supernatant was transferred to a 96-well plate in duplicate. The samples were mixed with the reaction cocktail and incubated for 15 min in the dark at room temperature prior to generating fluorescence readings on a Molecular Devices SpectraMax M2 microplate reader (Sunnyvale, CA) at Ex/Em 570/600 nm. Background H_2_O_2_ levels of *E. coli* ATCC 25922 incubated in dH_2_O were subtracted from reported results. A standard curve using different H_2_O_2_ concentrations was generated as a positive control and to quantify H_2_O_2_ concentration in experimental samples.

### ROS Detection

Prior to ROS detection, cells were incubated in 1 mM dihydrorhodamine (DHR) for 30 min, washed in 0.85% saline solution, and suspended in the appropriate experimental medium. *E. coli* ATCC 25922 cells exposed to 5 mM diamide were used as a positive control for ROS production. A Cytomics FC 500 flow cytometer (Beckman Coulter, Inc., Brea, CA) fitted with a 488 nm excitation laser was used to measure ROS production in the intracellular environment during CB-L exposure. Exponential-phase *E. coli* (10^8^ CFU/ml) was exposed to CB-L and incubated at 37°C with gentle rotary mixing for the specified time. Fluorescence was detected with the flow cytometer using channel FL1 with a 525 nm bandpass filter. A Molecular Devices SpectraMax M2 microplate reader was used to measure ROS production in CB-L without cells and of the *E. coli* intracellular environment while in the presence of CB-L. For these experiments, diluted cultures of stationary-phase *E. coli* (10^7^ CFU/ml) were exposed to CB-L and incubated at 37°C with gentle rotary mixing for the specified time. Fluorescence was measured at an excitation of 485 nm and emission of 525 nm.

### Carbon, Chelator, and ROS Scavenger Supplementation

*E. coli* ATCC 25922 cells were grown to mid-logarithmic phase, diluted to a starting density of 10^7^ CFU/ml, and washed with sterile 0.85% saline. Glucose (40 mM), EDTA (10 mM), DMSO (1%), and thiourea (150 mM) were added to the antibacterial CB leachate and incubated for 1 h at room temperature prior to exposure to cells. Washed cell pellets were resuspended in dH_2_O or CB-L with and without the addition of glucose, EDTA, DMSO, or thiourea. At designated times, 100 µl aliquots were removed and appropriate 10-fold dilutions were performed to determine viable CFU/ml.

### ELISA to Detect 8-OHdG and Oxidatively-damaged Nucleic Acids

*E. coli* ATCC 25922 cells were grown to mid-logarithmic phase, diluted to a 40 ml volume at a density of 5×10^8^ CFU/ml, washed with sterile 0.85% saline, and resuspended in experimental conditions (dH_2_O, CB-L, 2 mM H_2_O_2_ in dH_2_O, 1% DMSO in dH_2_O, 1% DMSO in CB-L, 10 mM EDTA in dH_2_O, or 10 mM EDTA in CB-L). At the designated times, 100 µl aliquots were removed and appropriate 10-fold dilutions were performed to determine viable CFU/ml. In addition, 20 ml samples were removed, pelleted at 4,000 RPM for 10 min, and subjected to DNA extraction. To coat the wells, 100 µl aliquots of 0.1% poly-l-lysine were added to 96-well plates (Corning, high bind EIA) for 5 min at room temperature and removed via aspiration prior to subsequent experimental use. Plates were loaded with quadruplicate 50 µl samples of 100 ng/ml nucleic acids and allowed to dry overnight in a 37°C incubator. Once dry, the plates were incubated without agitation in blocking buffer (1×PBS, 1.5% FBS) for 1 h at 37°C and subsequently washed 3× with wash buffer (1×PBS, 0.5% Tween 20). 100 µl aliquots of the mouse anti-8-OHdG antibody (1∶20,000 in blocking buffer) were added to each well of the 96-well plate, and the plate was incubated without agitation for 1 h at 37°C. After washing 3× with wash buffer, 100 µl of rabbit anti-mouse enzyme-linked secondary antibody (1∶10,000 in blocking buffer) (Sigma Aldrich) was added to each well. Incubation was performed at room temperature for 1 h and subsequently washed 3× with wash buffer. Finally, to allow for color development, 100 µl of 4-methylumbelliferyl phosphate (Sigma Aldrich) solution was added to each well, and the plate was incubated without agitation for 30–60 min at 37°C. Fluorescence was measured with a microtiter plate reader (Molecular Devices SpectraMax M2) using wavelengths of 360 and 440 for excitation and emission, respectively.

### Protein Oxidation

*E. coli* ATCC 25922 cells were grown to mid-logarithmic phase, diluted to a 20 ml volume at a density of 1×10^8^ CFU/ml, washed with sterile 0.85% saline, and resuspended in either dH_2_O, 2 mM H_2_O_2_, or CB-L. At the designated times, 100 µl aliquots were removed and appropriate 10-fold dilutions were performed to determine viable CFU/ml. In addition, 10 ml samples of the culture were removed, pelleted at 4,000 RPM for 5 min, and resuspended in 500 µl of lysis buffer (10 mM HEPES, pH 7, 6% SDS). To collect cell extracts containing the total protein population, the pellets were resuspended in 500 µl of lysis buffer and boiled for 5 min. Carbonyl derivatization was completed according to methods described by Shacter et al. [28]. Briefly, 200 µl of 2,4-dinitrophenylhydrazine in 2 M HCl was added to 1 mg of protein, gently mixed, and incubated at room temperature for 30 min. Proteins were then precipitated by the addition of 200 µl of 20% trichloroacetic acid and pelleted at 13,000 RPM for 5 min. The pellets were washed three times in 1∶1 ethanol:ethyl acetate for 10 min each, dried for 10–20 min, and resuspended in 600 µl of 6M guanidine hydrochloride, 20 mM NaPO_4_, pH 8. Duplicate 200 µl samples were transferred to a 96-well plate, and the A_280_ and A_360_ readings were measured for each sample with a Molecular Devices Spectramax M2 spectrophotometer. Carbonyl concentration was calculated with a molar extinction coefficient of 22,000 M^−1^ cm^−1^. Reported carbonyl values were normalized across the samples based on the respective measured protein concentration.

### Lipid Peroxidation

Lipid peroxidation measurements were generated using the Lipid Peroxidation (MDA) Assay kit (Abcam, Cambridge, MA) according to the manufacturer's guidelines. Briefly, *E. coli* ATCC 25922 cells were grown to mid-logarithmic phase of growth, diluted to 10^8^ CFU/ml, and washed with sterile 0.85% saline prior to exposure to dH_2_O, 2 mM H_2_O_2_, or CB-L. At each time point, 400 µl of the sample was collected, pelleted, resuspended in the manufacturer's lysis buffer, and subjected to bead beating two times for 30-sec intervals. The lysates were pelleted, the supernatant was processed with thiobarbituric acid, and the fluorescent lipid peroxidation products were measured on a microtiter plate reader (Molecular Devices SpectraMax M2) using wavelengths of 532 and 553 for excitation and emission, respectively.

### Preparation of Metal Ion Stock Solutions and Metal Salt Mixtures

Metal ion stock solutions were prepared by adding chloride salts to a final concentration of 100 mM in dH_2_O. Two different synthetic microbicidal mixtures (SMM3 and SMM16) were prepared by diluting metal ion stocks in dH_2_O to the concentrations found in CB07-L [1]. Elemental chemistry of the CB leachates was previously measured by inductively coupled plasma mass spectrometry (ICP-MS) (described in Otto et al. [1]), and ion concentrations from these ICP-MS measurements were used to prepare SMM3: Li (Sigma), 7.62 µM; Be(II) (Sigma), 0.80 µM; Na (Fisher), 171.61 µM; Mg(II) (Sigma), 773.11 µM; Al(III) (Sigma), 1079.6 µM; Ca(II) (Sigma), 6911.18 µM; Ti(IV) (Sigma), 0.01 µM; V(III) (Sigma), 0.49 µM; Mn(II) (Sigma), 99.31 µM; Fe(III) (Sigma), 229.88 µM; Co(II) (Sigma) 3.38 µM; Ni(II) (Sigma), 3.46 µM; Cu(II) (Sigma) 3.18 µM; Zn (Sigma) 14.52 µM [1]. The pH of the solution was adjusted to 3.5 with 1 M NaOH, and the solution was sterilized by passage through a 0.22 µM filter prior to further use. SMM16 was prepared in an identical manner as described above with the following metal ion concentrations: Fe(III), 200 µM; Co(II), 4 µM; Cu(II), 80 µM; Zn, 40 µM.

### Statistical Analyses

Unpaired Student's t test and ANOVA were used to assess statistical significance. A *p* value of <0.05 was considered statistically significant.

## Results

### Low pH does not mediate clay antibacterial activity

**Figure 1:**
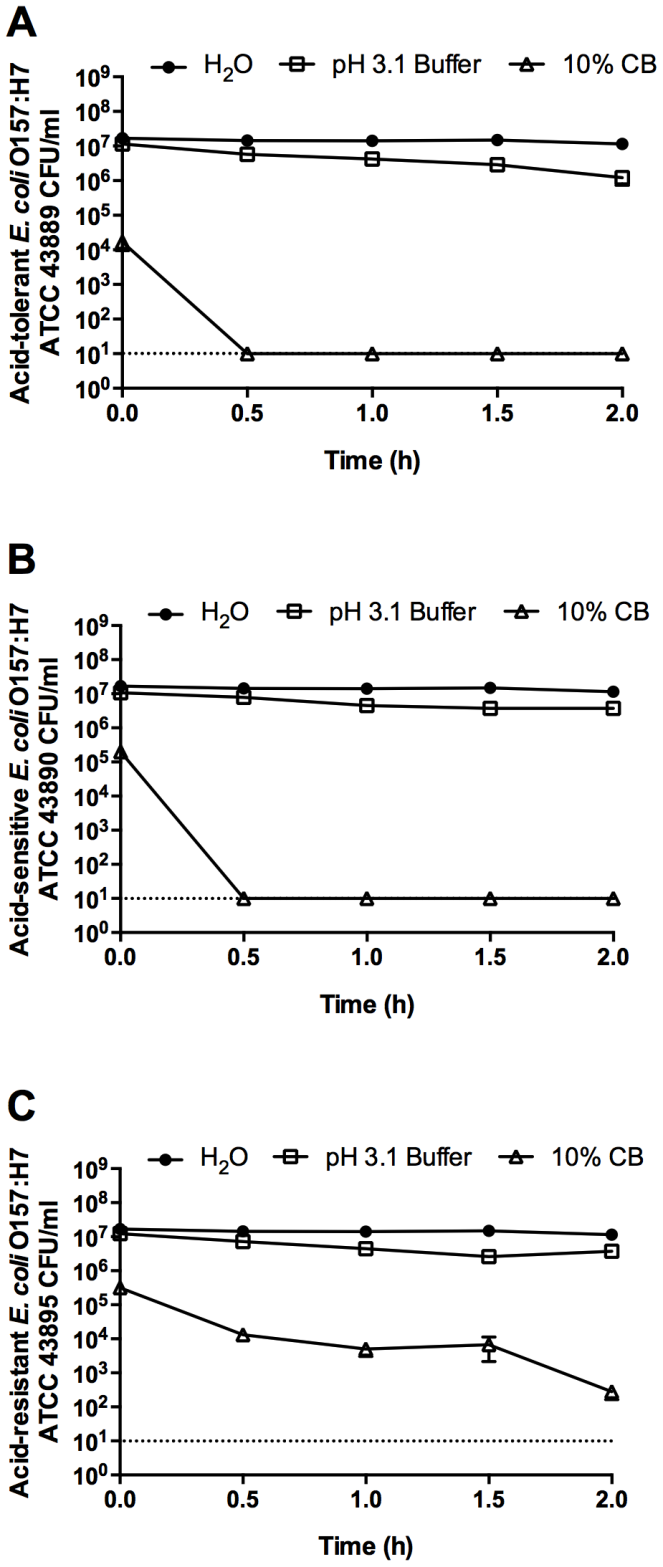
Stationary-phase, acid-tolerant (A), acid-sensitive (B), and acid-resistant (C) *E. coli* O157:H7 in 10% CB suspension (pH 3.1) and low-pH buffer (pH 3.1). Stationary-phase cells were grown overnight in LB media. Data are presented as the mean ± standard deviation (SD) for three independent experiments. The dotted line represents the limit of detection.

To determine the role of pH in the antibacterial activity of CB clay mixtures, we exposed three acid-responsive strains of *E. coli* O157:H7 (acid resistant, acid sensitive, and acid tolerant) to a 10% suspension of CB clay. The clay suspension completely killed the acid-tolerant and acid-sensitive *E. coli* O157:H7 strains (ATCC 43889 and 43890, respectively) within 30 min, whereas a low-pH buffer resulted in a minimal decrease in viability after 2 h (Fig. 1A, B). Time course killing curves demonstrated that the more adept acid-response systems in acid-resistant *E. coli* O157:H7 (ATCC 43895) significantly slowed the rate of CB-mediated killing and enhanced survival over 2 h (Fig. 1C).

**Figure 2:**
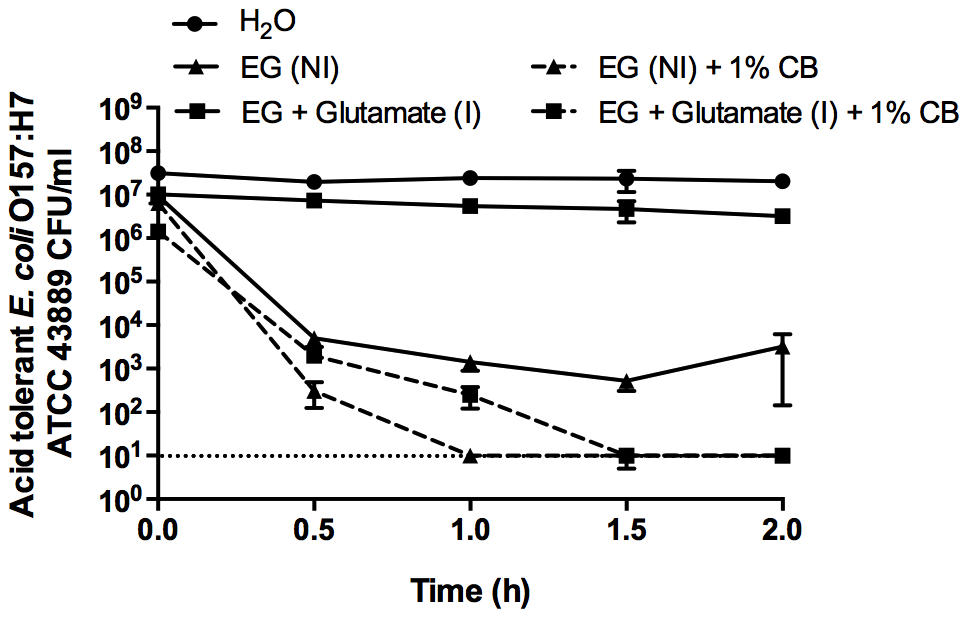
Stationary-phase, inducible-acid-tolerant *E. coli* O157:H7 challenged in EG medium (pH 2.5) with (I, induced) or without (NI, non-induced) 2 mM glutamate. Experimental samples were exposed to a 1% CB suspension for 2 h. Data are presented as the mean ±SD for three independent experiments. The dotted line represents the limit of detection.

Acid resistance was chemically induced in the acid-tolerant *E. coli* O157:H7 ATCC 43889 strain by supplementing the medium with glutamate during CB mineral exposure. Additionally, to eliminate cross protection between stationary-phase induced AR1 and glutamate-induced AR2, low pH experiments were also performed following overnight bacterial growth in glucose-supplemented medium which suppresses AR1 [11]. Induction of AR2 with glutamate significantly enhanced *E. coli* O157:H7 ATCC 43889 survival in the low pH EG medium (Fig. 2) Compared to a non-inducing (NI) environment with 1% CB, glutamate-induction of AR2 in acid-tolerant *E. coli* O157:H7 delayed the time required for the CB suspension to completely kill the cells (Fig. 2); however, after a 2 h exposure to 1% CB, chemically-induced, acid-resistant *E. coli* O157:H7 was completely killed (Fig. 2). Due to the rapid killing by CB-L, the viability of *E. coli* at the initial time point was often lower than the other control values. Further, we previously showed that when the pH of CB-L is increased, the killing activity is minimized [1]. Take altogether, these data (Figs. 1, 2) confirm that the low pH environment influences the rate at which CB clay mixtures kill, but does not directly mediate microbicidal activity.

### Hydrated antibacterial clays and derived leachates generate H_2_O_2_

**Figure 3:**
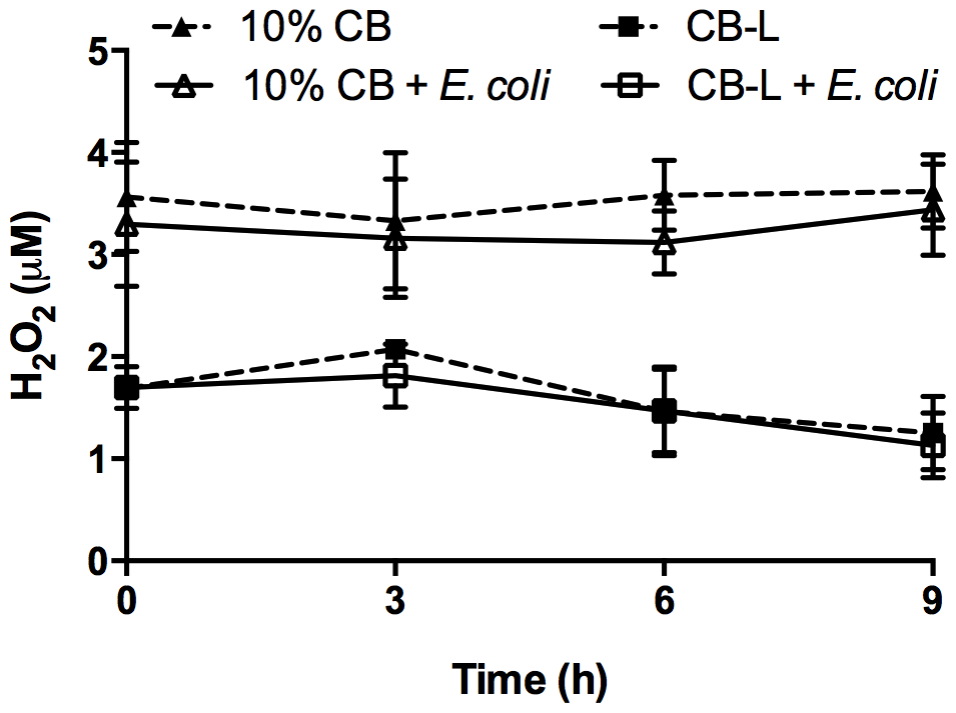
Hydrogen peroxide concentration in 10% CB suspensions and CB-L. Hydrogen peroxide levels were measured in 10% CB suspensions and CB-L with or without the addition of stationary-phase *E. coli* ATCC 25922. Values represent the average H_2_O_2_ concentration ±SD for three independent experiments.

We used a fluorescence detection system to measure H_2_O_2_ production in clay and leachate samples in the presence and absence of *E. coli* ATCC 25922 and observed continuous generation of 3–4 µM H_2_O_2_ in 10% suspensions of CB clay mixtures (Fig. 3). When the experimental medium was supplemented with up to 5 µM H_2_O_2_, the H_2_O_2_ was rapidly scavenged by the cells and was not detectable after 1 h (data not shown). H_2_O_2_ levels were slightly lower in CB-L with detected fluorescence correlating to 1–2 µM H_2_O_2_ when compared to a standard curve (Fig. 3). These lower H_2_O_2_ levels measured in CB-L could be due to the absence of CB clay mixtures required for surface-mediated production of H_2_O_2_ [29], [30]. Together, these data suggest that any H_2_O_2_ scavenged by the bacterial cells is rapidly regenerated by the hydrated CB clay mixtures or leachates, thus, resulting in a steady state concentration over time (Fig. 3). While there was continuous generation of 1–4 µM H_2_O_2_ in the clays and leachates, it is known that *E. coli* (strain AB1157) can produce and tolerate as much as 4 µM s^−1^ H_2_O_2_ during active growth [24].

### Hydrated antibacterial clays and derived leachates lead to intracellular and extracellular ROS

**Figure 4:**
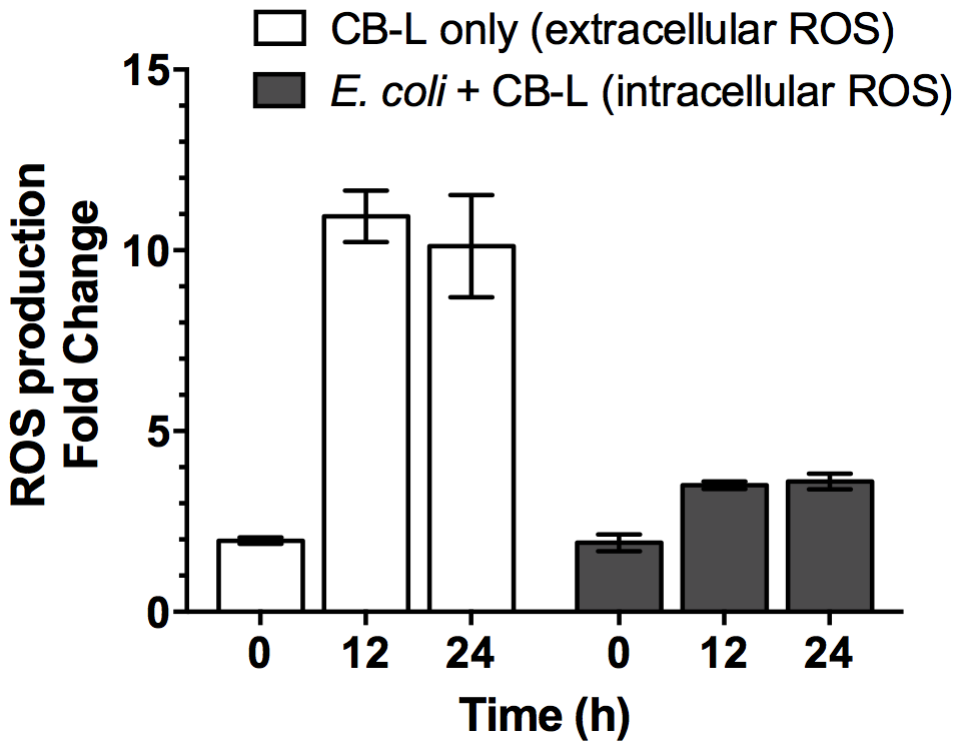
CB-L-associated ROS production in the absence and presence of *E. coli* ATCC 25922. ROS was measured in CB-L without the presence of cells (extracellular; using a plate reader) and exclusively in the intracellular environment of exponential-phase *E. coli* following CB-L exposure (intracellular; using flow cytometry). Cells were grown overnight, transferred to fresh growth media and grown to mid-logarithmic phase, washed, and exposed to CB-L for 24 h. Reported values represent the ROS production fold change of CB-L compared to water and CB-L-exposed cells compared to water-exposed cells. Data are presented as the mean ± standard error of the mean (SEM) for three independent experiments.

To determine the location of potential reactive oxygen toxicity, we assessed intracellular and extracellular ROS production. We measured 11-fold and 10-fold increases in ROS production in CB-L, as compared to a dH_2_O-only control, after 12 h and 24 h, respectively (Fig. 4). Following 12 h and 24 h CB-L exposures, flow cytometric analyses of *E. coli* ATCC 25922 cells revealed 3-fold and 2-fold increases in ROS production, respectively, as compared to the water-exposed control (Fig. 4). The oxidation-reduction potential (ORP) of CB10-L measured 593.3 mV [1], demonstrating that the solution is more oxidizing than the ultrapure, dH_2_O (432.6 mV) used in this study. Subsequent measurements over 24 h and thereafter revealed that the CB10-L ORP remains stable over time. Overall, these data demonstrated that CB-L generates an oxidizing environment and ROS. We note that our data conflicts with another study [31] which showed that the redox potential of antibacterial clay suspensions transitions to a reducing environment over time, however, the report did not include a methodological description for the ORP measurements.

### Antibacterial leachates induce lipid peroxidation lesions in *E. coli* ATCC 25922

**Figure 5:**
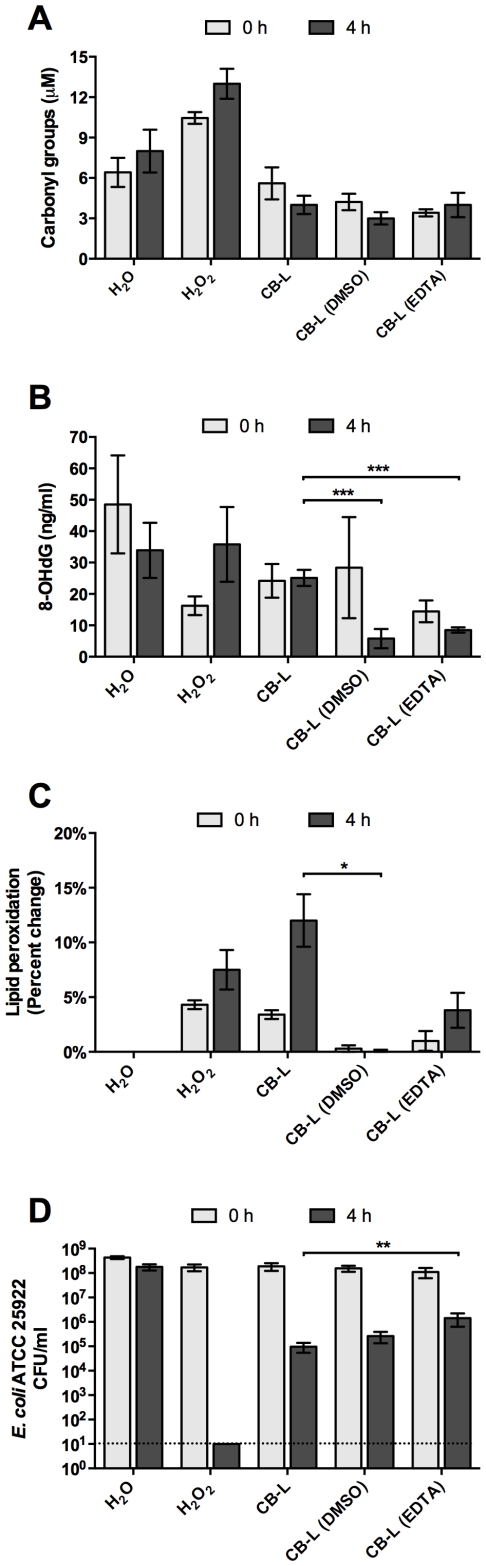
Assessment of *E. coli* ATCC 25922 oxidative damage to proteins (A), DNA (B), and lipids (C) and killing (D) upon exposure to CB-L and ROS scavengers or metal chelators. (A) Oxidative damage to protein samples collected from *E. coli* exposed to dH_2_O, H_2_O_2_ (2 mM), and CB-L with and without the addition of DMSO (1%) or EDTA (10 mM). Values represent the concentration of total carbonylated protein lesions formed as a result of protein oxidation during control and experimental exposures. Error bars represent the SEM for at least three independent experiments. (B) 8-OHdG levels measured in *E. coli* following CB-L exposure with and without the addition of DMSO or EDTA. Error bars represent the SEM for at least three independent experiments. (C) Lipid peroxidation levels measured in *E. coli* exposed to dH_2_O, H_2_O_2_, and CB-L with and without the addition of DMSO or EDTA. Values represent the percent change in lipid peroxidation over the water-exposed control, and the SEM for at least three independent experiments. (D) Viability of logarithmic phase *E. coli* following CB-L exposure with and without the addition of DMSO or EDTA. Error bars represent the SEM for at least three independent experiments. The dotted line represents the limit of detection. *, *p*<0.05; **, *p*<0.01; ***, *p*<0.002.

**Figure 6:**
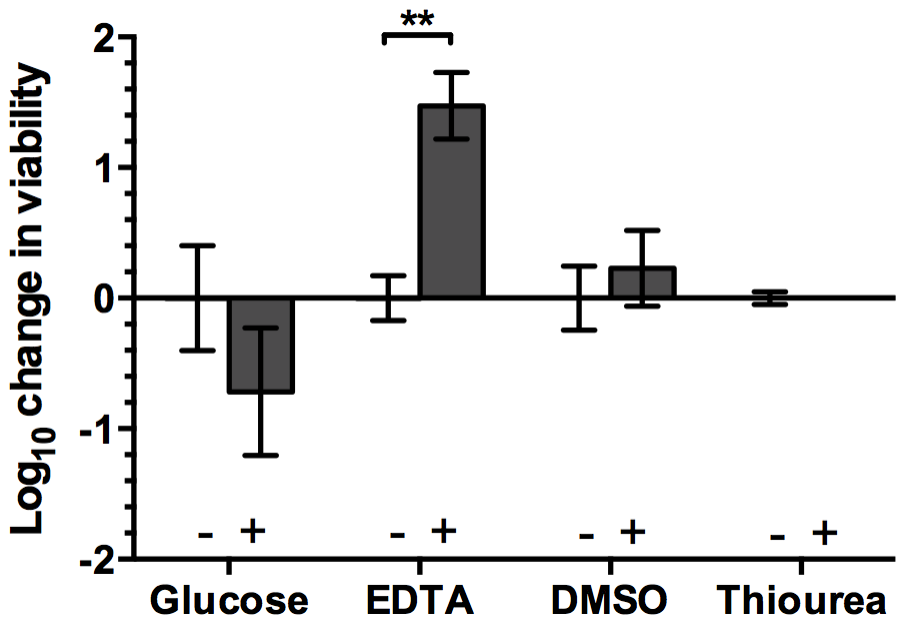
Comparison of log_10_ change in *E. coli* viability following exposure to CB-L with and without the addition of glucose (40 mM), EDTA (10 mM), DMSO (1%), and thiourea (150 mM). **, *p*<0.01.

We measured the production of oxidative lesions in DNA, proteins, and lipids following leachate exposure. First, we used a carbonyl-derivative-based biomarker assay to measure direct oxidation of amino acid side chains in total protein samples of *E. coli* following CB-L exposure. Compared to water-exposed controls, *E. coli* exposure to CB-L decreased protein oxidative lesions, although the difference was not statistically significant (*p* = 0.0604) (Fig. 5A). Furthermore, addition of DMSO or EDTA to CB-L did not significantly affect protein oxidative damage (Fig. 5A). While it is possible that the water-exposed cells undergo osmotic shock during this exposure, the repeatable minimal change in viability suggested that influences of osmotic shock are negligible. Next, we used an ELISA-based method to measure changes in 8-oxo-2'-deoxyguanosine concentrations (8-OHdG), a well-characterized biomarker for DNA lesions induced by oxidative stress [32]. Compared to water-exposed controls, no significant change in 8-OHdG was detected following *E. coli* exposure to CB-L for 4 h (Fig. 5B). However, the addition of DMSO (hydroxyl radical scavenger) or EDTA (broad-spectrum chelator) to CB-L prior to *E. coli* exposure significantly decreased oxidative DNA lesions (Fig. 5B). Finally, we used a malonaldehyde (MDA)-based assay to quantify the extent of lipid peroxidation in *E. coli* cells following CB-L exposure. Data from these experiments demonstrated that after exposure of *E. coli* to CB-L, lipid peroxidation significantly increased by 12% over the water-exposed control cells (Fig. 5C). In summary, CB-L mediated bactericidal activity (Fig. 5D, Fig. 6) and significantly increased lipid peroxidation, but did not induce DNA or protein oxidative damage (Fig. 5A, B).

### Supplementation with glucose does not rescue cells from clay-mediated killing

*E. coli* K-12 (strain JM101) metabolite profiling studies using ^13^C-glucose labeling demonstrated that conserved post-transcriptional rerouting of the central carbon metabolic fluxome may be instrumental in combating oxidative stress and works to counteract oxidative shifts in the cytoplasmic redox state [33]–[36]. Collectively, the metabolic redistribution inactivates specific enzymes in the glycolytic pathway resulting in an increased flux through the pentose phosphate pathway (PPP). Induction of the PPP results in increased production of nicotinamide adenine dinucleotide phosphate-oxidase (NADPH) which is essential in maintaining the function of key antioxidant systems and other cellular processes [33], [34], [36]. We supplemented the antibacterial CB clays with glucose and assessed bacterial viability following clay exposure. With the addition of glucose, CB-mediated killing was maintained (Fig. 6), indicating that the bacterial cells were rapidly killed via mechanisms other than oxidative stress.

### Supplementation with metal chelators rescues cells from clay-mediated killing

We supplemented CB-L suspensions with EDTA and assessed subsequent changes in *E. coli* ATCC 25922 viability. EDTA is a broad-spectrum chelator with high affinity for divalent and trivalent metal ions. The formation constants of EDTA-metal complexes vary depending on the pH of the solution and generally increase in affinity with decreasing pH [37]. EDTA is a hydrophilic molecule that does not penetrate cell membranes, thus, it will only chelate metals outside the cell. In a previous study, Jayasena et al. [38] exposed human neuroblastoma cells to Fe and H_2_O_2_ to induce hydroxyl radical stress. In these conditions, subsequent supplementation with EDTA did not significantly reduce hydroxyl radical production [38]. When CB-L was supplemented with EDTA, *E. coli* survival was significantly increased (*p* = 0.0094), indicating that the metals, not hydroxyl radicals, are killing the cells (Fig. 5D, Fig. 6). When CB-L was supplemented with DMSO, a hydroxyl radical scavenger, *E. coli* cells exhibited the same degree of killing as compared to CB-L alone (Fig. 5D, Fig. 6). Supplementation with DMSO during CB-L exposure eliminated lipid peroxidation (Fig. 5C). However, despite supplementation-based restoration of lipid integrity to levels comparable to the water-exposed control, CB-L maintained the same, or more, killing activity (Fig. 5D, Fig. 6). Further, when 150 mM thiourea, another hydroxyl radical scavenger, was added to the CB clay leachate or water immediately prior to contact with *E. coli* ATCC 25922, antibacterial activity remained unchanged, resulting in complete killing of *E. coli* within 24 h [3]. In summary, supplementation with ROS scavengers did not restore viability, whereas supplementation with metal chelators reduced CB-L-mediated killing.

### Clay leachate sustains antibacterial activity in an anoxic environment

**Figure 7:**
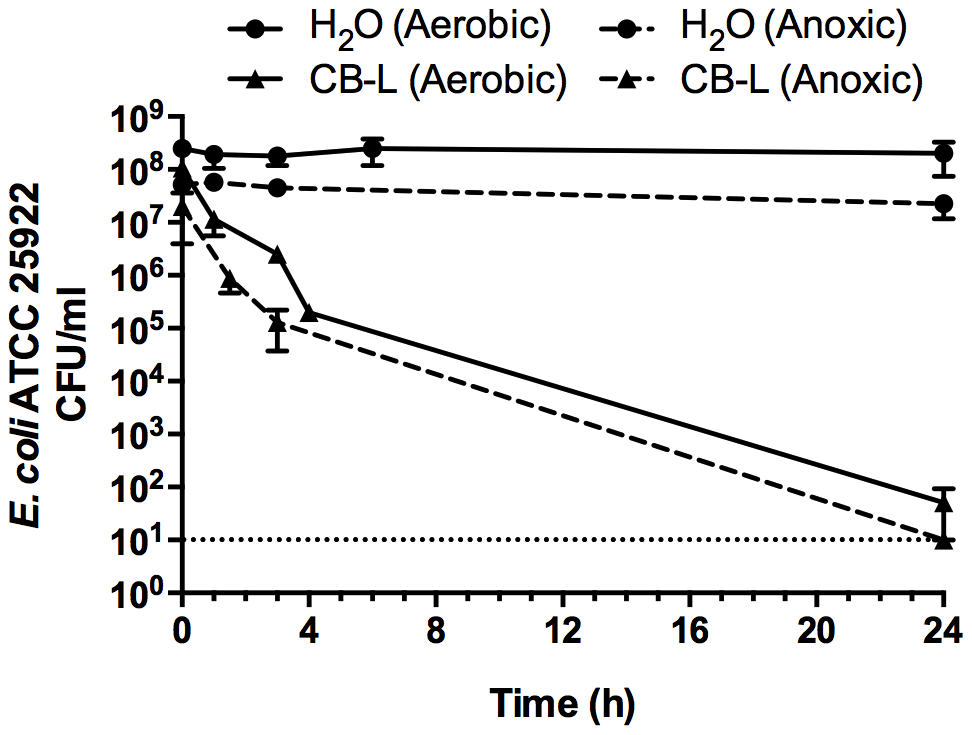
Assessment of *E. coli* viability during exposure to CB-L under aerobic and anoxic conditions. Error bars represent the SEM for three independent experiments. The dotted line represents the limit of detection.

We exposed *E. coli* to CB-L under anoxic conditions, whereby ROS are not formed due to the lack of molecular oxygen. Data from time-course exposures of *E. coli* ATCC 25922 to CB-L demonstrated parallel killing rates over 24 h, with complete killing occurring in the anoxic-exposed samples after 24 h (Fig. 7). Therefore, CB-L was equally lethal to cells in an anaerobic chamber as to cells incubated in an aerobic environment. Thus, reactive oxygen species were not required for lethal activity and did not contribute to toxicity of CB-L.

### Solutions of metal ion salts completely kill *E. coli* and MRSA

**Figure 8:**
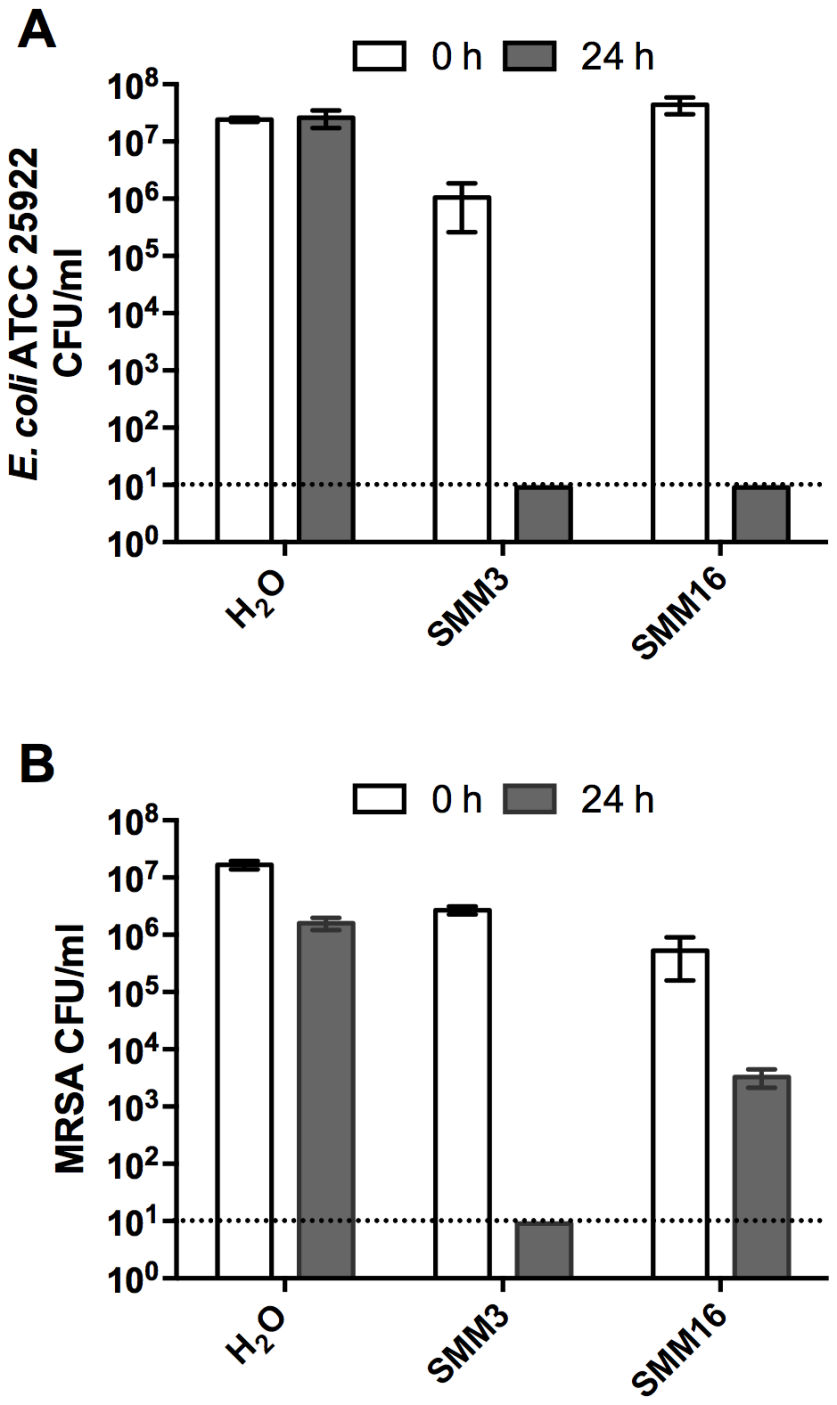
Assessment of *E. coli* ATCC 25922 (A) and MRSA (B) viability following exposure to SMM3 and SMM16 synthetic microbicidal mixtures of metal salts. The dotted line represents the limit of detection.

We previously used ICP-MS to analyze concentrations of metal cations present in the natural CB leachates [1]. Using the concentrations presented in Otto et al. [1], we prepared solutions of metal chloride salts, termed synthetic microbicidal mixtures (SMMs), that contained these metals at the same concentration and pH as in the natural leachate and tested these solutions for antibacterial activity against *E. coli* ATCC 25922 and MRSA. We should note, however, that the concentrations of metals in the natural leachates substantially vary [1] and, therefore, the metal concentrations in the SMMs only approximate those of natural leachates. SMM3, which was composed of all metals through Zn on the periodic table (see Materials and Methods for a complete list), completely killed *E. coli* and MRSA after a 24 h exposure (Fig. 8). Previous experiments in our lab demonstrated that *E. coli* exhibits increased survival in CB07-L supplemented with desferrioxamine [3], which readily chelates Fe, Co, Cu, and Zn [39]. Therefore, we prepared SMM16, which contained only Fe, Co, Cu, and Zn metal salts. The SMM16 solution completely killed *E. coli* and exhibited bactericidal properties against MRSA after a 24 h exposure (Fig. 8). These data support the conclusion that metal ions contribute to the antibacterial activity of the leachates.

## Discussion

Some metals are known to exhibit toxicity in humans and some bacterial species. Previous literature reports that redox-active and -inactive metals increase production of hydroxyl radicals, superoxide, or hydrogen peroxide [40]–[42] and deplete cellular antioxidant defenses, particularly thiol-containing antioxidants and enzymes [43]. The exhaustion of antioxidant defenses is hypothesized to lead to oxidative stress and subsequent oxidative lesions in cellular lipids, proteins, and DNA. The toxic effects of metal exposure are often attributed to these lesions, as supplementation with extrinsic antioxidants ameliorates damage to the cellular macromolecules [44]. Here, we corroborate these previous data by demonstrating that antibacterial CB clay mixtures and leachates exhibit a strongly oxidizing environment, as revealed by high oxidation-reduction potential values [1], release redox-active metal ions [1], and generate H_2_O_2_ and ROS. We tested the hypothesis that metals released from the antibacterial CB clays generate H_2_O_2_ and ROS, and, in turn, oxidatively damage cellular macromolecules leading to eventual death. However, we did not observe a significant increase in oxidatively-damaged proteins or DNA. These data are consistent with previous literature demonstrating that Cu-exposed *E. coli* did not exhibit oxidatively-damaged DNA [45]. While Cu-supplemented cells exhibited substantial hydroxyl radical formation, the authors showed that the majority of H_2_O_2_-oxidizable Cu was located in the periplasm. Therefore, most of the Cu-mediated hydroxyl radical formation occurs in this compartment and away from the DNA [45]. While copper has previously been shown to cause oxidative damage to DNA and proteins, the experiments were conducted with purified DNA and protein samples and, thus, may not be relevant for living cell systems [46].

Supplementation of the leachate with thiourea and DMSO (ROS scavengers) failed to rescue the cells from killing. While catalase and superoxide dismutase are commonly used ROS scavengers, the enzymes were not used in this study because the low pH and high metal content of the leachates and clay suspensions would cause rapid loss of enzymatic activity. Rather, we supplemented the antibacterial CB leachate with thiourea and DMSO, which maintain ROS scavenging abilities in low pH environments [47], [48].

Exposure of *E. coli* ATCC 25922 to clay and leachate under anoxic conditions resulted in complete killing, demonstrating that oxygen was dispensable for toxicity. Iron toxicity is often explained by its participation in the Fenton reaction (Fe^2+^ + H_2_O_2_ → Fe^3+^ + HO· + OH^−^) [5], [49], [50]; however, this mechanism depends on the presence of O_2_ and thus cannot account for anaerobic toxicity [51].

Peroxidative damage of lipids correlated with high levels of extracellular ROS, but did not cause lethality. Recent reports suggest that classic antibiotics kill bacteria by indirect formation of ROS [52]. However, additional studies revealed that antibiotic exposures do not produce ROS and that lethality likely resulted from the direct inhibition of cell wall assembly, protein synthesis, and DNA replication [53]. These latter data are analogous to our data whereby oxidative stress is secondary to the direct mode of killing.

To our knowledge, this work is the first analysis of a complex antibacterial metal ion mixture with a strong oxidizing environment [1]. Previous literature addressing individual metals provides insight into the possible mechanism of action of the CB leachate, although the mechanism likely involves a sequence of events that are more complex than those in systems only containing one type of toxic metal ion. For example, Cd exposure increases the generation of ROS during exposure to bacterial cells [54], [55]. However, Cd is not a redox-active metal, so the mechanism of superoxide generation was unclear. Wang et al. [54] and Pacheco et al. [56] demonstrated that Cd interferes with cellular respiration by directly binding to complexes II and III in the electron transport chain in liver, brain, and heart mitochondria of guinea pig. When the function of complex III is inhibited in mitochondria, superoxide is generated, leading to further site-specific damage; however, the oxidative damage is not the principle mechanism of Cd toxicity [54], [57], [58]. Pacheco et al. [56] demonstrated that fermenting *E. coli* K-12 cultures, grown in media streamed with nitrogen and capped with a rubber stopper, are less sensitive to Cd toxicity than actively respiring cultures. Wang and Crowley [59] reported that Cd affects *E. coli* K-12 expression of genes associated with protein synthesis, energy metabolism, and cell rescue following 5, 10, and 25 min exposures. The up-regulation of genes associated with anaerobic metabolism and the shutdown of all high-energy consumption processes such as the biosynthesis of amino acids suggests that, upon exposure to Cd, cells switch to an energy conservation mode [59]. In a similar manner, the mechanism of action of Ag has been linked to its interaction with thiol group compounds such as those found in the respiratory enzymes of bacterial cells [60]–[63].

In this report, we corroborate the historical correlation between oxidative stress and metal exposure. However, we negate the hypothesis that the oxidative environment, generated by antibacterial clays and leachates, is required for killing. We collectively conclude that clay-mediated killing is not due to oxidative damage, but rather via toxicity associated directly with released metal ions. Our ongoing transcriptomics and molecular genetics studies will be used to investigate direct molecular markers of oxidative and metal stress and define the molecular mechanism of action. A comparison of expression profiles from cells exposed to the leachates under oxic versus anoxic conditions will also be important to assist in differentiating between the mechanism of toxicity in these two environments.
